# High-Fat and Low-Carbohydrate Diets Are Associated with Allergic Rhinitis But Not Asthma or Atopic Dermatitis in Children

**DOI:** 10.1371/journal.pone.0150202

**Published:** 2016-02-26

**Authors:** So Young Kim, Songyong Sim, Bumjung Park, Jin-Hwan Kim, Hyo Geun Choi

**Affiliations:** 1 Department of Otorhinolaryngology-Head & Neck Surgery, Seoul National University Hospital, Seoul, Korea; 2 Department of Otorhinolaryngology-Head and Neck Surgery, CHA Bundang Medical Center, CHA University, Seoul, Korea; 3 Department of Statistics, Hallym University, Chuncheon, Korea; 4 Department of Otorhinolaryngology-Head & Neck Surgery, Hallym University Sacred Heart Hospital, Anyang, Korea; 5 Department of Otolaryngology–Head and Neck Surgery, Hallym University Sacred Heart Hospital, Seoul, Korea; University of Kansas Medical Center, UNITED STATES

## Abstract

**Background:**

Numerous studies have suggested that nutritional intake is related to allergic diseases. Although conflicting results exist, fat intake is often associated with allergic diseases. We investigated the relationship between allergic diseases and nutritional intake after adjusting for various demographic and socioeconomic factors in a large, representative sample of Korean children.

**Methods:**

A total of 3,040 participants, aged 4 to 13 years old, were enrolled in the present study from the Korean National Health and Nutrition Examination Survey (KNHANES), 2010–2012. Nutritional intake data, including total calories, protein, fat, carbohydrate, vitamin A, vitamin C, thiamine, riboflavin, and niacin, were retrieved from the survey using the complete 24-hour recall method. The associations between each nutritional factor and allergic rhinitis/asthma/atopic dermatitis were analyzed using simple and multiple logistic regression analyses with complex sampling. Age, sex, body mass index (BMI), number of household members, income level, and region of residence were adjusted for as covariates.

**Results:**

Of the participants, 22.1%, 6.0%, and 15.5% suffered from allergic rhinitis, asthma, and atopic dermatitis, respectively. Allergic rhinitis was significantly correlated with high-fat and low-carbohydrate diets. The adjusted odds ratio (AOR) was 1.25 (95% CIs = 1.06–1.46, P = 0.007) for fat intake, denoting a 10% increase. Carbohydrate intake (10% increase) was negatively related to allergic rhinitis with an AOR of 0.84 (95% CIs = 0.74–0.95, P = 0.004). No other significant relationships were found between the retrieved nutritional factors and either asthma or atopic dermatitis.

**Conclusion:**

Allergic rhinitis was related to high-fat and low-carbohydrate diets. Although the underlying mechanisms and causal relationships remain elusive, the present study provides reliable evidence regarding the associations between nutritional factors and allergic rhinitis by considering numerous factors within a large and representative population.

## Introduction

Allergic diseases, including allergic rhinitis, asthma, and rhinoconjunctivitis, are common chronic diseases. The united airways disease hypothesis suggests that allergic rhinitis and asthma are related through a single inflammatory process [1]. The prevalence and associated factors of respiratory allergic diseases, along with other allergic diseases (e.g., atopic dermatitis) have often been considered together as aspects of common allergic immune reactions.

Although variations exist in the study designs and population characteristics regarding the prevalence of allergic diseases, approximately 12–14% (and up to 25–40% of children) are estimated to suffer from asthma, allergic rhinitis, and atopic dermatitis worldwide [2–4]. The prevalence of allergic diseases showed a noticeable increase over last few decades of the late 20^th^ century, especially in developed countries [5,6]. Although the prevalences of many allergic diseases, including atopic dermatitis and asthma, have shown no substantial increase [7] or even decreased, the prevalence of food allergies is still increasing [8], and many allergic diseases continue to increase in developing countries where the prevalence had been very low in the past [9]. Urbanized environmental and dietary factors likely increase this prevalence. For instance, several studies have found a possible link between allergic diseases and dietary factors [10–13]. Specifically, previous studies have demonstrated that fat intake and subsequent obesity are related to asthma, allergic rhinitis, and rhinoconjunctivitis [14]. Excessive fat consumption is one of the notable characteristics of the modern westernized diet pattern because of the high intake of fast food, meats, and confections. Therefore, the high-fat diet of modern life might partially account for the recent increased prevalence of allergic diseases. Because most previous research has provided only cross-sectional or epidemiologic evidence, the causal mechanisms relating fat intake and allergic diseases remain elusive; however, certain immunologic roles of fat might mediate the relationship between these conditions [14].

Although numerous studies have suggested that nutritional factors are related to allergic diseases, only a few studies with a considerable risk of bias have suggested associations between low vitamin A, D, and E levels and high total polyunsaturated fat intake with asthma and/or wheezing [15]. Other than asthma, allergic diseases, such as allergic rhinitis and atopic dermatitis, have not been examined in this regard. Moreover, few studies have concurrently analyzed multiple nutritional factors including both macro- and micronutrients. Therefore, we simultaneously investigated the relationship between allergic diseases and numerous nutritional factors. Moreover, much of the past research has been limited with conflicting results most likely because of the confounding effects of covariates and heterogeneous study populations. The present study attempted to minimize these limitations by adjusting for numerous possible confounders within a large, representative sample.

## Materials and Methods

### Sample and Data Collection

The Institutional Review Board of the Korea Centers for Disease Control and Prevention approved this study (2010-02CON-21-C, 2011-02CON-06-C, and 2012-01EXP-01-2C). Permission via written informed consent was obtained from the parents or guardians of each participating child prior to the survey.

This study employed a cross-sectional design that used data from the Korea National Health and Nutrition Examination Survey (KNHANES), which includes information from the entire nation. All of the data are available from the KNHNES database (https://knhanes.cdc.go.kr/knhanes/index.do), which is freely available to researchers who agree to conform to ethical research principles. The survey includes a health interview, a nutritional survey, and physical examinations. Statistical methods were applied based on the sampling design using adjusted weighted values. The Centers for Disease Control and Prevention of Korea collected the KNHANES data; the data collected from 2010 to 2012 were analyzed. Within each of those years, a panel selected 192 districts. Within those districts, 20 households were sampled to represent the entire Korean population. The surveys evaluated data from the civilian, non-institutionalized South Korean population using a stratified, multistage, clustered sampling method based on national census data. The statisticians who performed the post-stratification weighted the sample and accounted for both non-response rates and extreme values.

**Fig 1:**
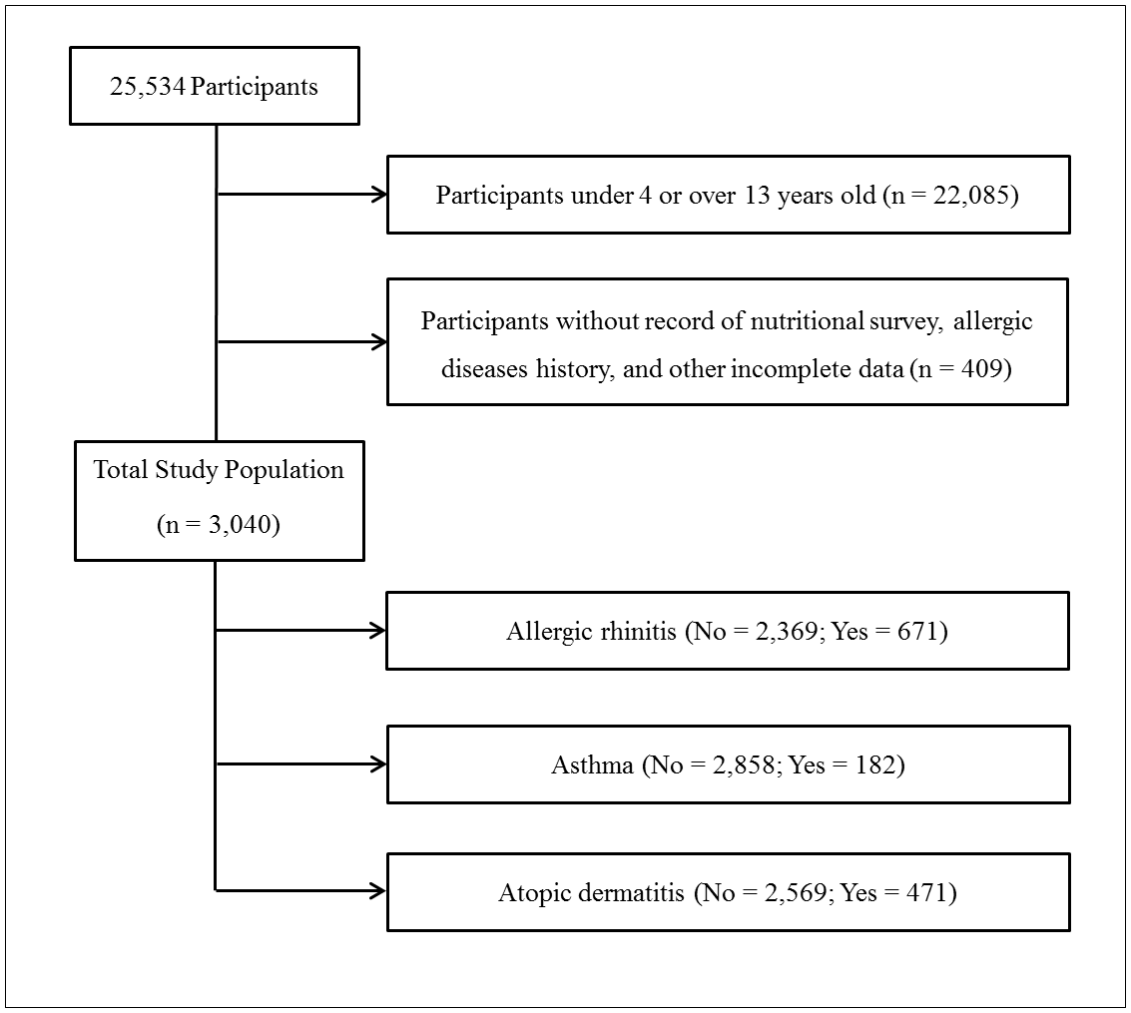
A flow sheet of participant selection in the present study. Among a total of 25,534 participants, participants under 4 or over 13 years old or participants without record of nutritional survey, allergic diseases history, and other incomplete data was excluded. Resultant 3,040 participants were comprised of 2,369 participants with allergic rhinitis, 2,858 participants with asthma, and 2,569 participants with atopic dermatitis.

Of the 25,534 total participants, we excluded participants who were under 4 years old or over 13 years old (22,085 participants); did not perform the nutritional survey; or had no record of allergic rhinitis, asthma, atopic dermatitis, the number of household members, income level, or region of residence (409 participants). A total of 3,040 participants (1,609 male; 1,431 female, aged 4 to 13 years old) comprised the final sample (Fig 1).

### Survey

Trained staff collected food intake data using the complete 24-hour recall method. Answers on specific days (e.g., holidays or weekends) were not included. The intake amounts of total calories (kcal/day), total protein (g/day), vitamin A (μg RE/day), vitamin C (mg/day), thiamine (mg/day), riboflavin (mg/day), and niacin (mg/day) were calculated by referencing the nutrient concentrations in foods according to the Korean Food Composition Table [16]. The intake amount was compared with the recommended dietary intake amount for Korea [17]. Therefore, the proportions of total calories, total protein, vitamin A, thiamine, riboflavin, and niacin for each participant were calculated after adjusting for age and sex. For example, the proportion of total intake calories (%) = total intake calories/age and sex matched the recommended total intake calories. The intake of protein was calculated as the proportion of the age- and sex-matched recommended intake amount for individuals in Korea. However, no recommended intake amounts exist for fat and carbohydrates, and their intake in proportion to total intake calories is more important than the amount of intake. Unlike other nutritional components, balancing among protein, fat and carbohydrates is the most important factor regarding fat and carbohydrate intake measurements [17]. Thus, fat and carbohydrates were measured as the distribution of intake calories in total intake calories rather than the proportion of age- and sex-matched recommended intake. The proportions of fats and carbohydrates consumed were measured using the following methods: fat intake calories/total intake calories (%), and carbohydrate intake calories/total intake calories (%).

Body mass index (BMI, kg/m^2^) was categorized into 4 groups according to the Centers for Disease Control and Prevention guidelines regarding BMI for children and adolescents [18]: obesity = BMI ≥ 95^th^ percentile; overweight = BMI ≥ 85^th^ percentile and < 95^th^ percentile; healthy weight = BMI ≥ 5^th^ percentile and < 85^th^ percentile; and underweight = BMI < 5^th^ percentile. After dividing household income by the square root of the number of household members, monthly income was divided into 4 quartiles from top to bottom: low, low middle, upper middle, and high. The regions of residence were divided into 2 groups: urban (Seoul, Gyeonggi, Busan, Daegu, Incheon, Gwangju, Daejeon, and Ulsan) and rural (Gangwon, Chungbuk, Chungnam, Jeonbuk, Jeonnam, Gyeongbuk, Gyeongnam, and Jeju). The participants were asked about their histories of allergic rhinitis, asthma, and atopic dermatitis. For example, “Have ever been diagnosed with allergic rhinitis by a medical doctor?” was inquired. Participants with histories of medical diagnoses were recorded as positive.

### Statistical Analysis

To represent the entire Korean population, each surveyed population was weighted for specific value according to factors such as age, sex, and region of residence. Differences in mean age, the number of household members and the nutritional factors between the control group and the allergic rhinitis/asthma/atopic dermatitis group were compared using a linear regression analysis with complex sampling. The rate of differences regarding sex, BMI group, income level, and region of residence were compared using the chi-square test with the Rao-Scott correction.

The associations between each nutritional factor and allergic rhinitis/asthma/atopic dermatitis were analyzed using simple logistic regression analysis with complex sampling (unadjusted) and multiple logistic regression analysis with complex sampling adjusted for age, sex, BMI, the number of household members, income level, and region of residence (full-adjusted). Two-tailed analyses were conducted, and *P*-values lower than 0.05 were considered to indicate significance. The adjusted odds ratio (AOR) and 95% confidence intervals (CIs) for each nutritional factor were calculated. The odd ratios of nutritional factors were expressed as 10% increases. After applying the weighted values recommended by the KNHANES, all of the simple and multiple logistic regression analysis results were presented as weighted values. The results were analyzed using SPSS ver. 20.0 (IBM, Armonk, NY, USA).

## Results

In totals, 22.1% (671/3,040), 6.0% (182/3,040), and 15.5% (471/3,040) of the participants reported a diagnosis of allergic rhinitis, asthma, and atopic dermatitis, respectively, made by a physician (Table 1). The study included 1,948 participants without any type of allergic diseases; 887 participants had one type of allergic disease, while 178 and 27 participants had two and three types of allergic diseases, respectively. The age, sex, and income level significantly differed across the groups with regard to the presence of allergic rhinitis, asthma, and atopic dermatitis, respectively. Other socioeconomic factors such as the number of household members and region of residence did not significantly differ across groups with regard to the presence of allergic diseases (Table 1). Among the various nutritional factors, the amounts of protein, fat, carbohydrates, thiamine, and niacin significantly differed with regard to the presence of allergic rhinitis (Table 2).

**Table 1:** General Characteristics by Allergic Rhinitis, Asthma, and Atopic Dermatitis.

Characteristics	Allergic Rhinitis			Asthma			Atopic Dermatitis		
	No	Yes	P-value	No	Yes	P-value	No	Yes	P-value
Number (n‡, %)	2,369 (77.9)	671 (22.1)		2,858 (94.0)	182 (6.0)		2,569 (84.5)	471 (15.5)	
Age (year, mean, SD)	8.8 ± 0.1	9.0 ± 0.1	0.237	8.9 ± 0.1	8.3 ± 0.3	0.031*	8.9 ± 0.1	8.7 ± 0.2	0.505
Sex (n, %)‡			0.05			0.009†			0.109
Male	1,234 (50.6)	375 (56.1)		1,488 (51.1)	121 (62.7)		1,365 (52.5)	244 (47.4)	
Female	1,135 (49.4)	296 (43.9)		1,370 (48.9)	61 (37.3)		1,204 (47.5)	227 (52.3)	
BMI (kg/m^2^, n, %)‡			0.153			0.679			0.357
Underweight	103 (4.3)	30 (2.8)		128 (4.1)	5 (2.8)		109 (3.8)	24 (5.2)	
Healthy	1,629 (69.4)	482 (71.4)		1,991 (70.0)	120 (67.2)		1,792 (70.3)	319 (67.0)	
Overweight	383 (16.2)	107 (17.8)		457 (16.4)	33 (18.2)		410 (16.1)	80 (18.9)	
Obesity	254 (10.2)	52 (8.0)		282 (9.6)	24 (11.9)		258 (9.9)	48 (8.9)	
Household (N, mean, SD)	4.3 ± 0.0	4.2 ± 0.1	0.071	4.3 ± 0.0	4.3 ± 0.1	0.634	4.3 ± 0.0	4.3 ± 0.1	0.575
Income (n, %)‡			0.004†			0.284			0.574
Lowest	201 (11.7)	40 (8.7)		224 (11.0)	17 (12.4)		209 (11.5)	32 (8.7)	
Low Middle	710 (32.9)	160 (25.5)		813 (31.4)	57 (31.1)		740 (31.2)	130 (31.9)	
Upper Middle	812 (31.6)	260 (39.8)		1,023 (33.9)	49 (26.0)		900 (33.0)	172 (35.5)	
Highest	646 (23.7)	211 (26.1)		798 (28.3)	59 (30.5)		720 (24.3)	137 (23.9)	
Region (n, %)‡			0.586			0.427			0.196
Urban	1,690 (70.1)	479 (71.7)		2,038 (70.3)	131 (73.8)		1,829 (69.8)	340 (73.9)	
Rural	679 (29.9)	192 (28.3)		820 (29.7)	51 (26.2)		740 (30.2)	131 (26.1)	

* Mean difference using a linear regression analysis with complex sampling. Significance at P < 0.05.

^†^ Chi-square test with Rao-Scott correction and complex sampling. Significance at P < 0.05.

^‡^ The number of participants was described as unweighted values; Rate (%) was described as weighted values.

**Table 2:** Analysis of Nutritional Factors According to the Presence of Allergic Rhinitis, Asthma, and Atopic Dermatitis.

Nutritional Factors	Allergic Rhinitis			Asthma			Atopic Dermatitis		
	No	Yes	P-value	No	Yes	P-value	No	Yes	P-value
Total Calories (%, mean, SD)	68.1 ± 0.8	70.8 ± 1.3	0.069	68.6 ± 0.7	70.2 ± 2.2	0.479	68.4 ± 0.8	70.0 ± 1.9	0.401
Protein (%, mean, SD)	200.1 ± 2.7	212.9 ± 4.8	0.013*	202.1 ± 2.6	213.9 ± 8.6	0.192	202.8 ± 2.6	202.9 ± 5.9	0.980
Fat (%, mean, SD)	21.4 ± 0.2	22.8 ± 0.4	0.003*	21.7 ± 0.2	21.5 ± 0.8	0.758	21.9 ± 0.2	20.9 ± 0.5	0.068
Carbohydrates (%, mean, SD)	64.9 ± 0.3	63.3 ± 0.5	0.002*	64.5 ± 0.3	64.7 ± 1.1	0.884	64.4 ± 0.3	65.5 ± 0.6	0.095
Vitamin A (%, mean, SD)	124.9 ± 4.0	139.6 ± 9.8	0.128	125.2 ± 3.5	172.8 ± 29.1	0.093	129.6 ± 4.7	118.8 ± 5.7	0.128
Vitamin C (%, mean, SD)	121.5 ± 3.9	127.7 ± 6.1	0.359	123.2 ± 3.7	116.7 ± 9.0	0.509	123.0 ± 3.9	122.0 ± 6.3	0.889
Thiamine (%, mean, SD)	149.0 ± 2.4	159.3 ± 4.5	0.034*	151.0 ± 2.3	152.9 ± 7.4	0.809	151.4 ± 2.3	150.0 ± 4.6	0.770
Riboflavin (%, mean, SD)	130.9 ± 1.9	136.9 ± 4.0	0.161	131.8 ± 1.8	138.3 ± 6.4	0.308	131.7 ± 1.9	134.6 ± 3.8	0.487
Niacin (%, mean, SD)	126.1 ± 1.6	139.1 ± 3.1	<0.001*	128.7 ± 1.6	131.3 ± 5.4	0.639	128.5 ± 1.6	130.9 ± 3.8	0.534

* Mean difference according to a linear regression analysis with complex sampling, significance at P < 0.05.

Each nutritional factor was analyzed with regard to its relationship to allergic rhinitis, asthma, and atopic dermatitis. The strongest associations were found between allergic rhinitis and fat/carbohydrates (Table 3). As fat intake increased by 10%, allergic rhinitis significantly increased, with ORs of 1.27 (95% CI = 1.09–1.48, P = 0.002) and 1.25 (95% CIs = 1.06–1.46, P = 0.007) for the unadjusted and full-adjusted model, respectively. However, a 10% increase in carbohydrates was significantly associated with a decrease in allergic rhinitis, with ORs of 0.83 (95% CI = 0.74–0.93, P = 0.002) and 0.84 (95% CIs = 0.74–0.95, P = 0.004) for the same models, respectively. Slight but significant associations were observed between allergic rhinitis and protein (AOR = 1.02, 95% CIs = 1.00–1.03, P = 0.008), thiamine (AOR = 1.02, 95% CIs = 1.00–1.03, P = 0.035), and niacin (AOR = 1.03, 95% CIs = 1.01–1.05, P = 0.001). Vitamin A was associated with asthma (AOR = 1.01, 95% CI = 1.00–1.02, P = 0.014). No other nutritional factor was related to asthma or atopic dermatitis.

**Table 3:** Odd ratios for the nutritional factors regarding allergic rhinitis, asthma, and atopic dermatitis using a multiple logistic regression analysis with complex sampling.

	Allergic Rhinitis			Asthma			Atopic Dermatitis		
	OR	95% CI	P-value	OR	95% CI	P-value	OR	95% CI	P-value
Total Calories (10%)									
Unadjusted	1.03	1.00–1.07	0.065	1.02	0.97–1.08	0.468	1.02	0.97–1.07	0.394
Full-adjusted†	1.03	0.99–1.07	0.115	1.01	0.96–1.07	0.733	1.02	0.97–1.07	0.419
Protein (10%)									
Unadjusted	1.01	1.00–1.02	0.008*	1.01	1.00–1.03	0.157	1.00	0.99–1.01	0.980
Full-adjusted†	1.02	1.00–1.03	0.008*	1.00	0.99–1.02	0.639	1.00	0.99–1.01	0.909
Fat (10%)									
Unadjusted	1.27	1.09–1.48	0.002*	0.96	0.71–1.28	0.761	0.84	0.69–1.02	0.073
Full-adjusted†	1.25	1.06–1.46	0.007*	0.96	0.71–1.29	0.774	0.83	0.68–1.01	0.063
Carbohydrate (10%)									
Unadjusted	0.83	0.74–0.93	0.002*	1.02	0.78–1.33	0.884	1.14	0.97–1.34	0.103
Full-adjusted†	0.84	0.74–0.95	0.004*	1.02	0.78–1.33	0.901	1.15	0.98–1.35	0.091
Vitamin A (10%)									
Unadjusted	1.01	1.00–1.01	0.066	1.01	1.00–1.02	0.009*	1.00	0.99–1.00	0.181
Full-adjusted†	1.00	1.00–1.01	0.149	1.01	1.00–1.02	0.014*	1.00	0.99–1.00	0.165
Vitamin C (10%)									
Unadjusted	1.00	1.00–1.01	0.352	1.00	0.98–1.01	0.538	1.00	0.99–1.01	0.889
Full-adjusted†	1.00	1.00–1.01	0.364	0.99	0.98–1.01	0.297	1.00	0.99–1.01	0.842
Thiamine (10%)									
Unadjusted	1.02	1.00–1.03	0.029*	1.00	0.98–1.03	0.806	1.00	0.98–1.01	0.772
Full-adjusted†	1.02	1.00–1.03	0.035*	1.00	0.97–1.02	0.653	1.00	0.99–1.01	0.917
Riboflavin (10%)									
Unadjusted	1.01	1.00–1.03	0.131*	1.01	0.99–1.04	0.284	1.01	0.99–1.03	0.483
Full-adjusted†	1.02	1.00–1.04	0.056	1.01	0.99–1.04	0.392	1.00	0.99–1.02	0.659
Niacin (10%)									
Unadjusted	1.03	1.02–1.05	<0.001*	1.01	0.98–1.03	0.630	1.01	0.99–1.03	0.528
Full-adjusted†	1.03	1.01–1.05	0.001*	1.00	0.97–1.03	0.873	1.01	0.99–1.03	0.458

* Significance at P < 0.05.

^†^ Adjusted for age, sex, BMI, the number of household members, income level, and region of residence.

We conducted a subgroup analysis by age group and sex (S1 Table). According to the results, the prevalence of allergic rhinitis was significantly associated with fat (AOR = 1.30, 95% CI = 1.07–1.58, P = 0.009) and carbohydrate intake (AOR = 0.84, 95% CI = 0.72–0.98, P = 0.024) in the older age group (≥ 9 years old). In the female group, significant relationships were found between the prevalence of allergic rhinitis and the intake of protein (AOR = 1.03, 95% CI = 1.01–1.04, P = 0.005), fat (AOR = 1.37, 95% CI = 1.09–1.71, P = 0.006), and carbohydrates (AOR = 0.76, 95% CI = 0.64–0.91, P = 0.002).

## Discussion

We identified significant relationships between allergic rhinitis and both high-fat and low-carbohydrate diets among Korean children; however, no significant correlation was found between nutritional factors and asthma/atopic dermatitis. The present study was stronger than others given that we concurrently considered various nutritional factors including total calories, protein, fat, carbohydrates, vitamin A, vitamin C, thiamine, riboflavin, and niacin by adjusting their effects. These comprehensive considerations of various nutritional factors allowed the investigation of the adjusted contribution of each nutritional factor to allergic diseases, in contrast to previous studies, in which only a limited number of variables were considered [19–21]. In addition, several demographic and socioeconomic factors were adjusted to improve the reliability of the results.

Fat intake was significantly and positively related to allergic rhinitis in the present study. Similar to our results, previous studies have demonstrated positive relationships between fat intake and allergic rhinitis and asthma [14,19–21]. Although no direct evidence can explain the causal relationship between fat consumption and allergic diseases, several immunologic and epidemiologic studies have come to this conclusion. Certain types of fatty acids play an essential role in immune reactions. For instance, linoleic acid is a precursor of arachidonic acid, which is subsequently converted to prostaglandin E2 (PGE2) [1]. PGE2 activates and augments the allergic immune reaction by promoting the synthesis of immunoglobulin E (IgE). PGE2 inhibits the formation of interferon-r (IFNr) but not that of interleukin-4 (IL-4) in T-lymphocytes. Because IL-4 promotes (whereas IFN-r interferes with) the synthesis of IgE, the net effect of linoleic acid is an increase in the formation of IgE, which in turn enhances allergic reactions. Consistent with these findings, it has been suggested that *trans* fatty acids are associated with the prevalence of childhood asthma and allergies, likely due to their influence on the desaturation and chain elongation of n-6 and n-3 fatty acids into inflammatory mediators including prostaglandins and leukotrienes, thereby impairing immune reactions and contributing to atopic disease [22,23]. In fact, it has been observed that infants with increased *trans* fatty acid plasma levels show changes in the fatty acid composition of plasma lipids similar to those of atopic patients [24,25].

On the other hand, carbohydrates were negatively related to allergic diseases in the present study. To our knowledge, no prior study has found a relationship between carbohydrate consumption and allergic diseases in humans; however, some animal studies provide clues regarding the effect of carbohydrates on allergic diseases. The early supplements of specific oligosaccharides before and during pregnancy significantly mitigate acute allergic skin responses and lung resistance but increase regulatory T-cell concentrations, thereby leading to decreased sensitization and allergies in mice [26,27]. The plausible mechanism for these immune modulations away from the allergic reaction remains somewhat elusive; however, specific carbohydrate epitopes, such as galactose-a-1,3-galactose and galacto-oligosaccharides, can induce acute allergic reactions during ingestion [28]. Although carbohydrates are rarely immunogenic compared with protein, they can induce allergic reactions through cross-reactive carbohydrate determinants or specific epitopes [29]. Based on the hygiene hypothesis, less exposure to carbohydrates might have made our participants susceptible to carbohydrate allergies because of the lack of immunity to these epitopes [30].

Several types of nutritional factors, including protein, thiamine, and niacin, showed slight but significant correlations with allergic rhinitis. These proteins or micronutrients might play unknown but essential roles in allergic reactions. The very large sample sizes in the present study enabled us to detect these small but statistically significant effects at the population level. However, the average intakes of these nutrients, including protein, thiamine, and niacin, were over 100% in both the allergic and non-allergic groups, which implies that few or no participants had deficiencies with regard to these nutrients in the present study. Therefore, our results might not assess the effects of the low intake of these nutrients. Further studies based on children with deficiencies of each nutrient will delineate clear associations between these nutrient factors and allergic diseases.

Although asthma, atopic dermatitis, and allergic rhinitis are related to allergic immune reactions, the first two conditions did not manifest any relationship with the studied nutritional factors. This lack of a relationship might explain why allergic rhinitis was more prevalent than asthma and atopic dermatitis and potentiate statistical power in the present study. Furthermore, various factors other than the allergic immune response might influence the pathogenesis of asthma and atopic dermatitis. Because asthma is a lower airway disease, many factors other than allergic immune reactions might contribute to its pathogenesis. For instance, wheezing in children is associated with various respiratory diseases, especially respiratory viruses such as respiratory syncytial virus (RSV) [31]. Atopic dermatitis is an allergic skin disease caused by cutaneous barrier malfunction and inflammation as well as the interaction of the allergic immune system and skin microorganisms [32]. Compared with these allergic diseases, allergic rhinitis is an upper airway disease without lower airway or cutaneous pathophysiology effects.

The present study provided valuable epidemiologic evidence demonstrating the significant correlations between allergic rhinitis and both high-fat and low-carbohydrate diets by considering many nutritional, demographic, and socioeconomic factors in a large and representative sample of Korean children. Moreover, we confined the age groups of the children, who have different physiological characteristics than adults. We also conducted subgroup analyses by age. Although the statistical significance values were weakened by the lower AORs and broader 95% CIs, the significant associations between high fat and low carbohydrate intake and the prevalence of allergic rhinitis were maintained in the older age (≥ 9 years old) and female groups (S1 Table). The attenuation of these statistical significance values might be due to the limited study population resulting from the subgrouping strategy. However, the present study has some unavoidable limitations. Its cross-sectional study design was unable to determine a causal relationship between allergic diseases and nutritional factors. Furthermore, although we considered numerous confounders in our models, others such as home environment, lifestyle, and exercise habits might remain [33]. In terms of independent variables, we concurrently analyzed numerous nutritional factors, thereby enhancing the value of the present study over others; however, we did not considered subtypes of fat (e.g., saturated or unsaturated fats). In addition, the KNHANES was based on retrospective self-reports; therefore, recall bias remains an issue. The presence of allergic diseases was surveyed without objective tests such as skin tests. Because the KNHNES database only contained serum IgE levels in participants older than 9 years in 2010, we could not objectively determine the presence of allergic diseases in younger children in the present study. Furthermore, the complete 24-hour recall method may better represent dietary habits on a population level than on an individual level. A dietary history or a semi-qualitative food frequency questionnaire would be a better method for assessing associations between diet and diseases. Nevertheless, the complete 24-hour recall method remains one of the most commonly used tools among retrospective nutritional surveys. Additional studies with prospective, longitudinal designs are warranted to address these problems.

## Conclusions

Allergic rhinitis was significantly correlated with both high-fat and low-carbohydrate diets among children. Although making thorough adjustments for numerous confounders in a large, representative sample reinforced the reliability of our results, these relationships must be considered with regard to the dietary habits and other characteristics of the population (i.e., Korean children). Because diet is a modifiable factor, the relationships between allergic rhinitis and both fat and carbohydrate intake likely have significant clinical implications, especially with regard to preventive medicine.

## Supporting Information

S1 Table Subgroup analyses of odd ratios of nutritional factors for allergic rhinitis using multiple logistic regression analysis with complex sampling (full-adjusted model) (DOCX)
