# Supplementary material for: High-Fat and Low-Carbohydrate Diets Are Associated with Allergic Rhinitis But Not Asthma or Atopic Dermatitis in Children
Source: PLoS One. 2016 Feb 26;11(2):e0150202. doi: 10.1371/journal.pone.0150202 (PMC4769275; doi:10.1371/journal.pone.0150202)
Supplement: S1 Table — (DOCX) [file pone.0150202.s001.docx]

S1 Table. Subgroup analyses of odd ratios of nutritional factors for allergic rhinitis using multiple logistic regression analysis with complex sampling (full-adjusted model)

|  |  | Allergic Rhinitis | | |
| --- | --- | --- | --- | --- |
|  |  | AOR | 95% CI | P-value |
| Young age (≤ 8 years old) | |  |  |  |
|  | Protein (10%) | 1.02 | 1.00-1.03 | 0.018* |
|  | Fat (10%) | 1.19 | 0.92-1.53 | 0.179 |
|  | Carbohydrate (10%) | 0.83 | 0.68-1.01 | 0.059 |
| Old age (≥ 9 years old) | |  |  |  |
|  | Protein (10%) | 1.01 | 0.99-1.02 | 0.262 |
|  | Fat (10%) | 1.30 | 1.07-1.58 | 0.009* |
|  | Carbohydrate (10%) | 0.84 | 0.72-0.98 | 0.024* |
| Male | |  |  |  |
|  | Protein (10%) | 1.01 | 0.99-1.02 | 0.321 |
|  | Fat (10%) | 1.16 | 0.94-1.42 | 0.174 |
|  | Carbohydrate (10%) | 0.91 | 0.77-1.07 | 0.240 |
| Female | |  |  |  |
|  | Protein (10%) | 1.03 | 1.01-1.04 | 0.005* |
|  | Fat (10%) | 1.37 | 1.09-1.71 | 0.006* |
|  | Carbohydrate (10%) | 0.76 | 0.64-0.91 | 0.002* |

* Significance at P < 0.05.
